# Geriatric core competencies for non-geriatricians and nurses: a scoping review

**DOI:** 10.1186/s12877-025-06806-8

**Published:** 2025-11-25

**Authors:** Deng-Chi Yang, Yung-Chen Yu, Yi-Hsuan Lee, Ruei-Ning Su, Wei-Hung Lin, Pei-Chun Lai

**Affiliations:** 1 Department of Geriatrics and Gerontology, National Cheng Kung University Hospital, College of Medicine, National Cheng Kung University, Tainan, Taiwan; 2 School of Medicine, College of Medicine, National Cheng Kung University, Tainan, Taiwan; 3 Department of Nursing, National Cheng Kung University Hospital, College of Medicine, National Cheng Kung University, Tainan, Taiwan; 4 Department of Nursing, Tainan Hospital, Ministry of Health and Welfare, Tainan, Taiwan; 5 Division of General Internal Medicine, Department of Internal Medicine, National Cheng Kung University Hospital, College of Medicine, National Cheng Kung University, 138 Sheng Li Road, Tainan, 704 Taiwan; 6 Education Center, National Cheng Kung University Hospital, College of Medicine, National Cheng Kung University, 138 Sheng Li Road, Tainan, 704 Taiwan

**Keywords:** Geriatric core competencies, Bloom’s taxonomy, Non-geriatricians, Nurses, Scoping review

## Abstract

**Background:**

There is a lack of geriatric core competencies for non-geriatricians and nurses. Our review aimed to identify and analyse the key concepts of the core competencies in these groups.

**Methods:**

A systematic search was conducted through July 2025 using five electronic databases: Medline, Embase, Cochrane Library, Ageline, and Education Resources Information Center. Studies related to geriatric core competencies, involving research populations such as physicians, residents, medical students, registered nurses, and nurse practitioners, and conducted via expert consensus or the Delphi method were included. We classified the core competencies using an integrated domain-based approach and aligned them using Bloom’s taxonomy framework.

**Results:**

From the ten studies included in the final review, we identified 257 geriatric core competencies distributed into 18 domains. The most frequently mentioned competencies for physicians, medical students, and nurses were disease and comorbidities, pharmacologic/medication management, and cognitive/dementia. These competencies fall under the “patient care” category defined by the Accreditation Council for Graduate Medical Education (ACGME). In terms of Bloom’s taxonomy, 59.14% (152/257) of the geriatric core competencies were classified as *Apply* in the cognitive process dimension, and 64.2% (165/257) were classified as *Conceptual* in the knowledge dimension.

**Conclusions:**

Our study highlights that the most emphasized core geriatric competencies in patient care are disease management, medication management, and cognitive problems. However, critical areas, such as interdisciplinary teamwork and addressing patient and caregiver needs, are significantly underrepresented. This gap necessitates a curriculum that fosters a holistic approach to geriatric education among healthcare professionals.

**Supplementary Information:**

The online version contains supplementary material available at 10.1186/s12877-025-06806-8.

## Background

Dr. Marjory Warren, recognised as the mother of British geriatric medicine, played a pivotal role in advocating geriatric medicine as a distinct specialty. She emphasized the importance of educating medical students and nurses about the diseases prevalent in old age [1]. This focus is increasingly relevant considering the unique characteristics and clinical challenges associated with older adults [2]. In geriatric populations, the clinical presentations often deviate from typical patterns observed in younger individuals. These presentations are frequently multifactorial, involve atypical symptoms, and require prompt and precise treatment to mitigate rapid deterioration, poor treatment outcomes, slow recovery, and heightened risks associated with both the disease and its treatment [3]. Therefore, the distinctiveness and significance of geriatric medicine are critical areas of focus in contemporary medical education and practice [4, 5].

Competency-based medical education (CBME) has emerged as the dominant paradigm in ongoing reform efforts [6]. CBME prioritises learner-centered approaches and is structured around the core competencies that learners are expected to develop [7]. In February 1999, the Accreditation Council for Graduate Medical Education (ACGME) proposed six general competencies: patient care, medical knowledge, interpersonal and communication skills, professionalism, practice-based learning and improvement, and system-based practices. These competencies provide a systematic framework for the development of curricula and assessment processes in medical education. The ACGME has also formulated milestones across various specialties and subspecialties, necessitating the creation of specific milestones within each competency. These milestones are organised developmentally, describing a continuum of physician abilities from novice or early medical students to expert clinicians, and emphasizing ongoing practice improvement. Entrustable Professional Activities (EPAs) further complement the framework. They are defined as units of professional work that require adequate knowledge, skills, and attitudes, and are independently executable, observable, and measurable.

The updated ACGME geriatric medicine Milestones 2.0 [8], released in July 2021, integrates the 76 curricular milestones set by the American Geriatrics Society (AGS) and Association of Directors of Geriatric Academic Programs [9], as well as 12 EPAs [10]. This updated version comprises 6 core competencies, 22 subcompetencies, and 38 milestones, with patient care and medical knowledge reflecting the unique educational needs of the different specialties [4]. The AGS also developed minimum competencies in geriatrics for medical students, which are structured around the critical 5Ms of geriatrics: mind, mobility, medication, multicomplexity, and matters most [11]. Core competencies in geriatric care for internal medicine and family medicine residents [12] cover seven domains: medication management, cognitive health, complex or chronic illnesses, palliative and end-of-life care, patient safety, care transition, and ambulatory care, with training programs for healthcare professionals such as pharmacists [13] and physical therapists [14]. With regard to outcomes, the European undergraduate curriculum in geriatric medicine offers a pertinent example. Of the 19 learning objectives derived from the European undergraduate curriculum in geriatric medicine and mapped to the contents of the undergraduate curricula, only four features were found in all curricula: patient respect, medication use, multidisciplinary teamwork, and acute inpatient and emergency care.

Despite these advancements, a gap remains in the provision of advanced-quality care to older adults by general healthcare professionals who lack foundational geriatric knowledge. Previous systematic reviews of geriatric competencies have primarily focused on geriatric competencies for undergraduate medical students, focusing on the teaching methodologies and content [5, 15]. However, there is a lack of clarity regarding the core competencies of non-geriatricians and nurses. Our review addresses this gap by identifying and analysing the key concepts of core geriatric competencies for these groups to lay a foundation for future competency-based interdisciplinary education in geriatrics.

## Methods

This scoping review follows the Preferred Reporting Items for Systematic Reviews and Meta-Analyses extension for Scoping Reviews framework [16]. This scoping review was registered with the Open Science Framework (Registration URL: https://osf.io/cxkhv). The research question guiding this scoping review was as follows: What are the geriatric core competencies for non-geriatricians and nurses, and how are they distributed in terms of frequency and cognitive/knowledge dimensions? The term “non-geriatricians” is used to denote medical students, residents, and practicing physicians. To address the latter part of this question, the identified competencies were categorized using Bloom’s revised taxonomy for the cognitive and knowledge dimensions.

### Search strategy and selection criteria

A systematic search was initially conducted in June 2022 and was updated in July 2025 using the following five electronic databases: Medline, Excerpta Medica database (Embase), Cochrane Library, Ageline, and Education Resources Information Center (ERIC). This scoping review included studies that (1) were related to geriatric core competencies, (2) involved research populations that included physicians, residents, medical students, registered nurses, or nurse practitioners, and (3) were conducted via expert consensus or the Delphi method because of the clear and detailed core competencies in these studies. Search terms were developed under the headings “Physician”, “Nurse”, “Geriatric”, and “Competencies”. Truncations and controlled vocabulary were also employed. The full search strategy is described in the Appendix. The exclusion criteria were as follows: (1) no full text available, (2) duplicates, and (3) non-English publications.

### Charting data

All records were imported into EndNote 20 (Clarivate Analytics, Philadelphia, PA), and duplicates were removed. Records were allocated sequentially to six independent authors from the research team, and their titles were screened. The full texts of the selected citations were assessed in detail according to the inclusion criteria by two researchers from our team (YDC and YCY). Any disagreements between researchers were resolved through discussion or with the help of an additional researcher (SRN).

We quantified and classified core competencies. Two researchers from our team (YCY and YHL) classified all the core competencies based on their content. Any discrepancies in the classification were resolved by discussion with a third researcher (YDC) until consensus was achieved. The included studies of core competencies used various classification methods. Some studies employed a tripartite classification of knowledge, skills, and attitudes, whereas others classified competencies into distinct domains. To create a cohesive framework, we classified all the core competencies obtained from the included studies using an integrated domain-based classification, as outlined in the literature. This approach allowed us to systematically organize and compare competencies across different sources.

The collected competencies were classified using Bloom’s taxonomic framework. Bloom’s taxonomy has been an important tool for classifying learning objectives based on levels of cognitive complexity since the 1950 s [17]. In 2001, the taxonomy was revised to reflect advances in the science of cognitive development [18]. According to Bloom’s taxonomy guidelines and previous studies, verbs were classified into six cognitive dimensions (*Remembering*, *Understanding*, *Applying*, *Analyzing*, *Evaluating*, and *Creating*) and four knowledge dimensions (*Factual Knowledge*, *Conceptual Knowledge*, *Procedural Knowledge*, and *Metacognitive Knowledge*). This classification process involved examining each verb in the competencies and identifying the corresponding categories in the taxonomy. This study was conducted by four researchers from our team (YDC, YCY, YHL, and SRN). Each researcher independently classified competencies into cognitive and knowledge dimensions. In instances where competencies encompassed a multitude of verbs, a meticulous examination of the objectives inherent in these competencies in conjunction with a thorough analysis of the primary action words was undertaken for classification. Following a thorough review, the research team reviewed the classification and reached a consensus on inconsistent categories.

## Results

### Study selection

**Fig. 1:**
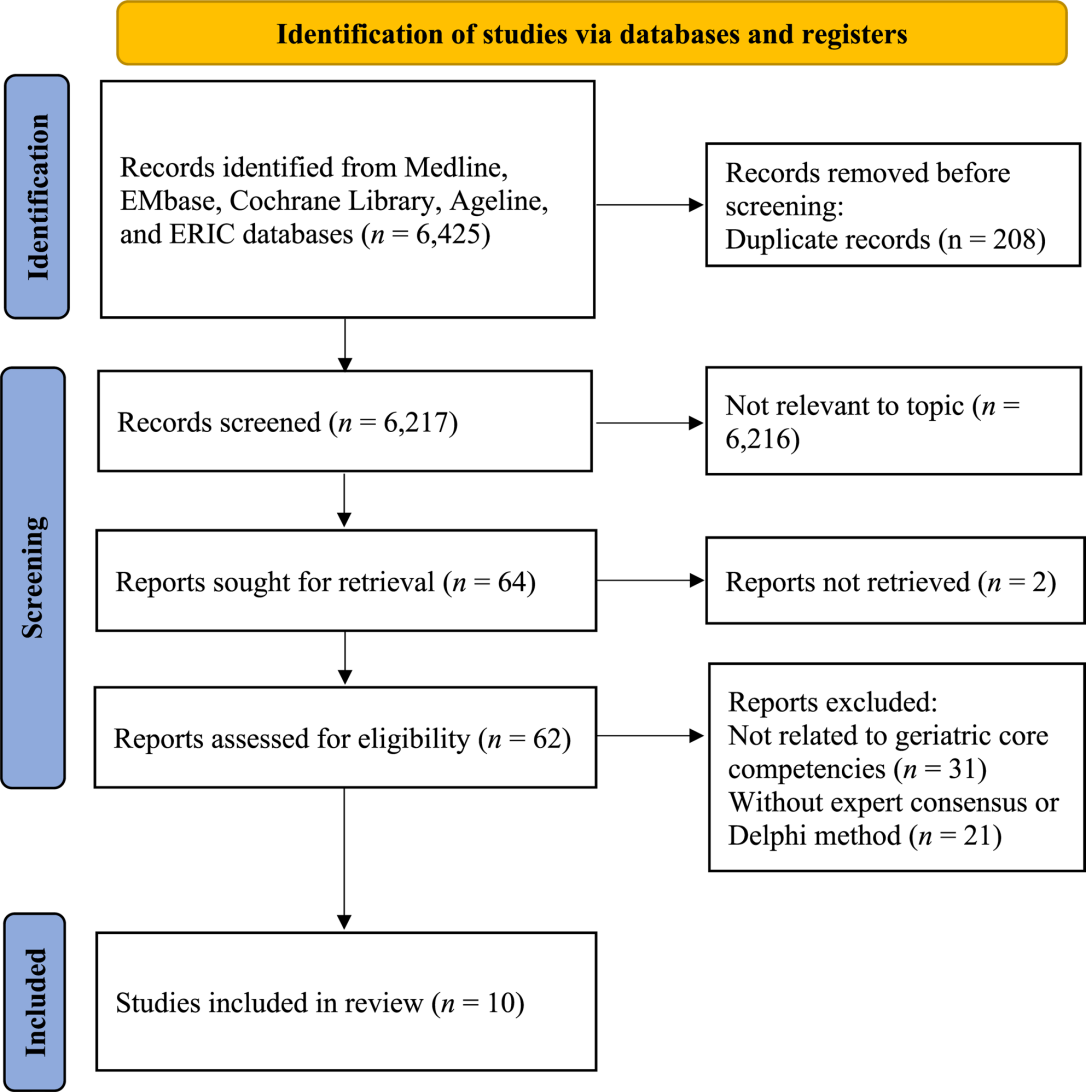
Flow chart of study selection

Records were identified through searches of electronic databases and reference lists (Fig. 1). Initially, 6,295 records were identified from the Medline (1,498 records), EMBASE (1,857 records), Cochrane Library (1,860 records), Ageline (934 records), and ERIC (146 records) databases. After removing 208 duplicates, 6,087 records were screened. Further screening for retrieval left 64 reports, 62 of which were assessed for eligibility. Reports were excluded if they were not related to geriatric core competencies (31 reports were excluded) or lacked expert consensus or the Delphi method (21 reports were excluded). A total of ten studies were included in the final review.

### Characteristics of included studies

Table 1 shows the details of the included studies, including the year of study, country, target group, expert group involved, number of experts, and outcomes. The studies were published between 1989 and 2017 and mostly involved American institutions, targeting groups that included medical and osteopathic students, nurses, and physicians. A total of 257 core competencies were identified. The outcomes ranged from six to 26 competencies. Generally, the development of core competencies is tailored to the educational requirements of healthcare professionals. The physicians had 20 competencies, while internal and emergency medicine residents each had 26. Family medicine residents were assigned 57 competencies for postgraduate year (PGY)I/II and 28 for PGY-III. Geriatric psychiatry fellowship included 16 competencies. Medical students had 26 competencies. Osteopathic medical students had six competencies. Nurses working in nursing or care homes were assigned 10–22 competencies.

**Table 1:** Characteristics of included studies

Studies	Year	Country	Target group	Expert group	Number of experts	Outcomes
Fox et al. [19]	1989	The United States of America	Geriatric medicine as a consultant physician	Feedback from 13 colleagues and initial consensus of 131 members	First round: 13 Second round: 131	20 competencies divided into *Knowledge*, *Attitude*, and *Skill*
The Education Committee Writing Group of the American Geriatrics Society [23]	2000	The United States of America	Medical and osteopathic students	AGS education committee	N/A	25 competencies divided into *Attitude* (7), *Knowledge* (14), and *Skill* (4)
Leipzig et al. [24]	2009	The United States of America	Medical students	Steering committee included people who had experience in developing competencies and in geriatrics and medical education, key stakeholders, including geriatricians, family physicians, general internists, neurology program directors, and general surgery program directors	13 members developed the Alpha Draft 315 participants ranked the draft of competencies	8 domains 26 competencies
Hogan et al. [20]	2010	The United States of America	Emergency medicine residents	Experts were invited from emergency medicine and Geriatric Medicine Association or Societies	Phase I: snow ball sampling, *n* = 363 Phase II: expert panel, *n* = 24	8 domains 26 competencies finalized as most relevant to EM
Williams et al. [12]	2010	The United States of America	Internal medicine and family medicine residents	Initial round: a group of academic educators and geriatricians from IM and FM who were involved in developing geriatric curriculums	Initial round: 8 academic educators Approximately 100 geriatric educators were divided into 8 working groups	7 domains 26 competencies
Mueller et al. [27]	2013	The United States of America	Nurses working in nursing homes	Nurse experts who were familiar with and engaged in nursing home culture change served as consultants	16	10 competencies
Noll et al. [26]	2013	The United States of America	Medical students	Osteopathic physician from ECOP Geriatricians	26	6 competencies
Charles et al. [21]	2014	Canada	PGY-I/II family medicine residents and PGY-III enhanced skills COE	Experts included individuals who participated in the Canadian Geriatric Society’s Core Competency Committee for medical students and the CFPC’s committees focused on geriatric care competencies and assessments for FM residents	6	57 for PGY-I/II family medicine residents, 28 for PGY-III COE residents
Swantek et al. [22]	2016	The United States of America	Geriatric psychiatry fellowship	American Association for Geriatric Psychiatry, the American Board of Psychiatry and Neurology, and the ACGME Psychiatry Residency Review Committee	N/A	16 competencies
Stanyon et al. [25]	2017	The United Kingdom	Registered nurses working in care homes	Medical and nursing professions, contributors to the British Geriatric Society	Round I: 26 Round II: 24 Round III: 20	22 competencies

*AGS* American Geriatric Society,*CFPC* College of Family Physicians of Canada, *COE* care of the elderly, *ECOP* Educational Council of Osteopathic Principles, *N/A* not applicable, *PGY* postgraduate year, *UK* United Kingdom, *USA* United States of America

To address our research questions, we consolidated core competencies into 18 distinct domains. This synthesis was achieved by compiling and analysing the domains of the ten studies included in our meta-analysis (Table 2). In our review, the target population for geriatric core competencies were consultant physicians, residents in subspecialties such as emergency, internal, and family medicine, medical students and subspecialty students, and nurses working in care homes and nursing homes. In five studies [12, 19–22] that focused on geriatric core competencies for physicians, the clinical sciences of aging, pharmacologic/medication management, cognitive/dementia problems, and transitions of care were consistently mentioned. The core competencies of medical students were disease, comorbidity, and optimising function, while those of nurses were teamwork, disease, comorbidity, and optimising function. Overall, the geriatric core competencies most frequently mentioned by physicians, medical students, and nurses were in the domains of disease and comorbidities, pharmacologic/medication management, and cognitive/dementia problems.

**Table 2:** Classification of geriatric core competencies into 18 domains

Reference	Fox et al. [19]	Hogan et al. [20]	Williams et al. [12]	Charles et al. [21]	Swantek et al. [22]	Education Committee of AGS [23]	Leipzig et al. [24]	Noll et al. [26]	Mueller et al. [27]	Stanyon et al. [25]	
Population	Consultant physician	EM residents	IM and FM residents	PGY-I/II FM residents	Geriatric psychiatric fellows	Medical and osteopathic students	Medical students	Medical students	Nurses working in nursing homes	Nurses working in care homes	
Domain of competencies											Total
1. Basic science of aging	+	-	-	-	-	+	-	-	-	-	2
2. Clinical science of aging	+	+	-	+	+	+	+	-	-	-	6
3. Social science of aging	-	-	-	-	-	+	-	-	-	-	1
4. Professionalism	+	-	-	+	+	+	-	-	-	+	5
5. Communication	+	-	-	+	+	-	-	-	+	-	4
6. Teamwork	+	-	-	-	+	+	-	-	+	+	5
7. Disease and comorbidity	-	+	+	-	-	+	+	-	+	+	6
8. Optimizing function	-	-	-	+	-	+	+	-	+	+	5
9. Geriatric assessment	+	-	-	-	+	+	-	-	-	+	4
10. Dealing with geriatric syndromes	-	-	+	+	+	+	-	-	-	+	5
11. Pharmacologic/medication management	+	+	+	+	-	+	+	-	-	+	7
12. Cognitive/dementia problems	+	+	+	+	-	+	+	-	-	+	7
13. Falling and gait abnormalities	-	+	-	-	-	-	+	+	-	-	3
14. Healthcare planning	+	-	-	+	+	-	+	-	-	+	5
15. Palliative care	-	-	+	-	-	-	+	-	-	+	3
16. Transitions of care	+	+	+	+	-	-	-	-	-	-	4
17. Patient safety and quality care	-	-	+	+	+	-	-	-	-	+	4
18. Research	+	-	-	-	-	-	-	-	+	-	2

*AGS* American Geriatric Society, *EM* emergency medicine, *FM* family medicine, *IM* internal medicine

### Data extraction and analysis using bloom’s revised taxonomy

**Fig. 2:**
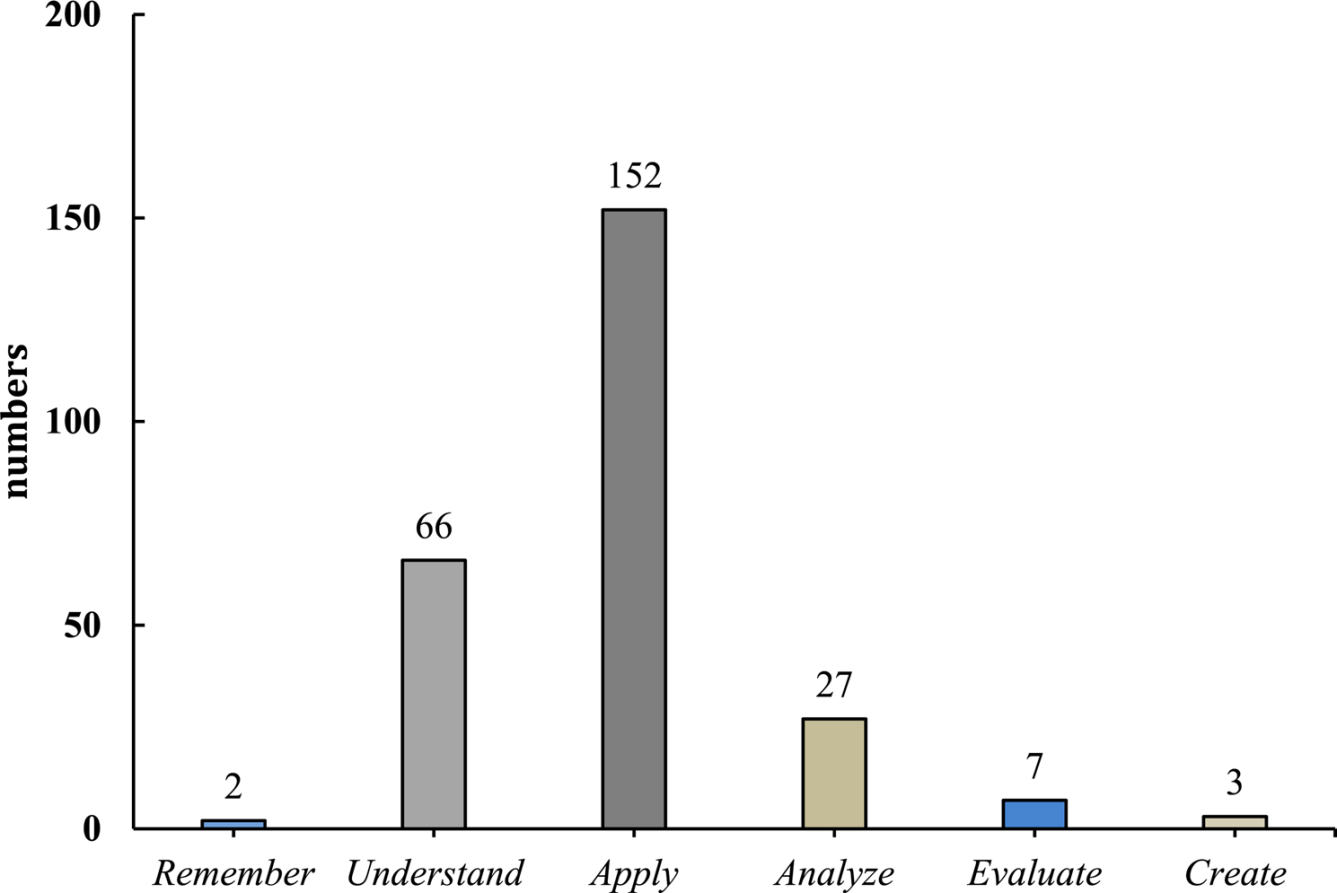
Geriatric core competencies classified using Bloom’s revised taxonomy for cognitive process dimension

**Fig. 3:**
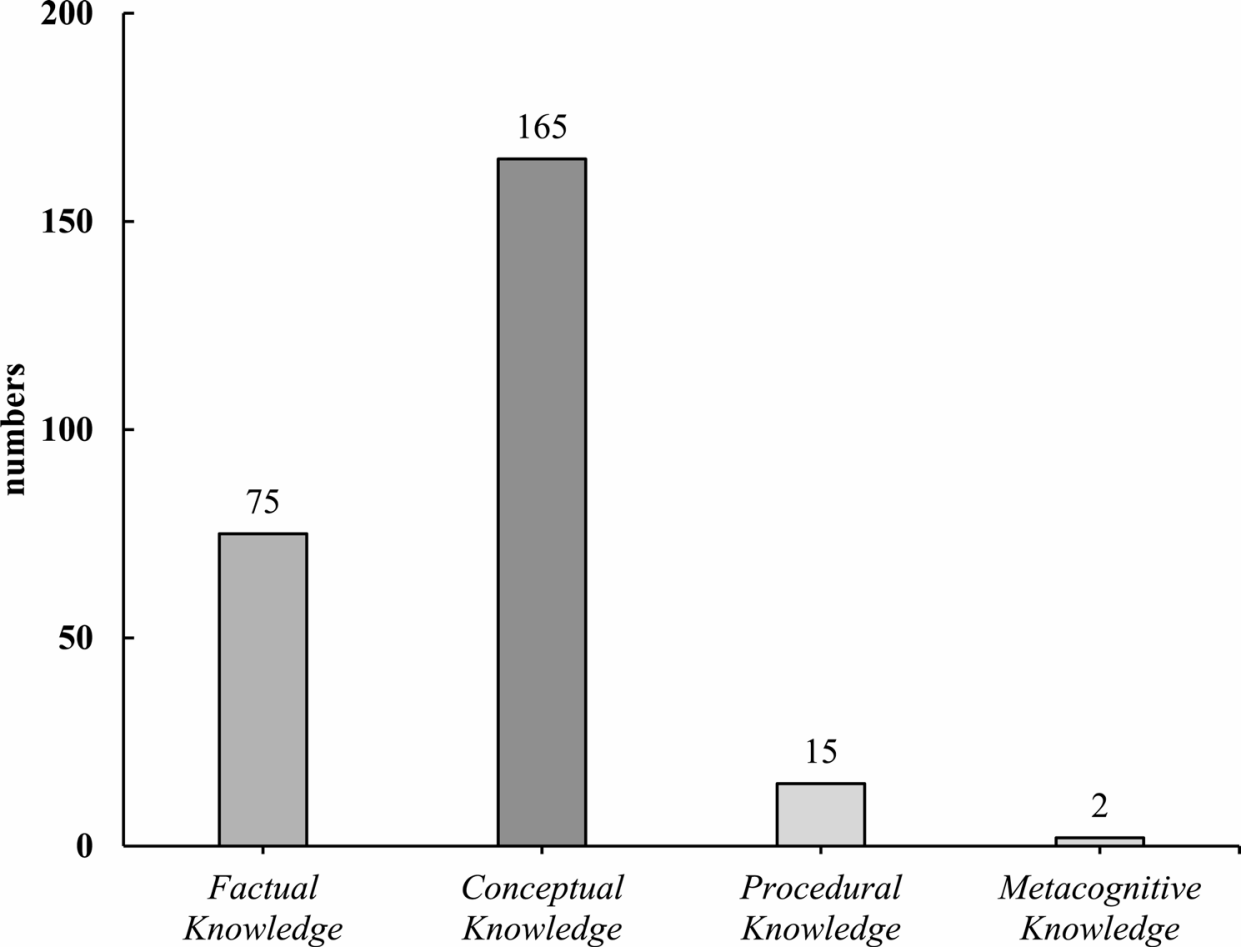
Geriatric core competencies classified using Bloom’s revised taxonomy for knowledge dimension

Figures 2 and 3 summarise the core geriatric competencies classified using Bloom’s revised taxonomy for the cognitive process and knowledge dimensions. For the cognitive process dimension, 59.14% (152/257) of the geriatric core competencies were classified as *Apply*, 25.68% (66/257) as *Understand*, and 9.73% (27/257) as *Analyze*. Only 2, 7, and 3 of the 257 competencies in the cognition process dimension were classified as *Remember*, *Evaluate*, and *Create*, respectively. Competencies classified as *Apply* were mentioned in eight studies, whereas those classified as *Understand* were mentioned in only two reports [22, 23].

Regarding the knowledge dimension, 64.2% (165/257) of the geriatric core competencies were classified as *Conceptual Knowledge*. The remaining were classified as *Factual Knowledge* (75/257), *Procedural Knowledge* (15/257), or *Metacognitive Knowledge* (2/257). Competencies classified as *Conceptual Knowledge* were mentioned in eight studies, whereas those classified as *Factual Knowledge* were mentioned in only two reports [22, 23].

## Discussion

This study employed a scoping review methodology to explore core competencies in geriatric medicine. Of the ten studies we reviewed, three focused on medical students, two on internal medicine/family medicine residents, one on emergency medicine residents, one on geriatric psychiatrists, one on consultant physicians, and two on nurses. From these studies, we identified 257 core geriatric competencies distributed across 18 domains. These competencies are essential for medical students, physicians, and nurses in providing geriatric care. These domains encompass a variety of areas, including the basic, clinical, and social sciences of ageing, professionalism, communication, teamwork, disease and comorbidity, optimising function, geriatric assessment, dealing with geriatric syndromes, pharmacologic/medication management, cognitive/dementia problems, falling and gait problems, healthcare planning, palliative care, transitions of care, patient safety and quality, and research.

Our analysis revealed that the most frequently cited geriatric core competencies in the literature were related to pharmacologic/medication management and cognitive/dementia problems, as evidenced by their mention in seven of the ten reviewed studies [12, 19–21, 23–25]. Furthermore, we observed differences in the core competencies required by physicians, medical students, and nurses as indicated by their frequency of mention in these studies. For physicians [12, 19–22], the essential domains of geriatric core competencies are the clinical science of ageing, pharmacologic/medication management, cognitive/dementia problems, and care transitions. For medical students [23, 24, 26], they are disease and comorbidity and optimizing function. For nurses [25, 27], they are teamwork, disease and comorbidity, and optimizing function.

In relation to the six general competencies endorsed by ACGME in February 1999, the competencies for physicians in the clinical science of ageing fall under “medical knowledge”, and those in pharmacologic/medication management, cognitive/dementia problems, and transitions of care fall under “patient care”. For medical students, all identified core competencies were associated with “patient care”. Notably, the most frequently cited core competencies for both physicians and medical students aligned with subspecialty-specific milestones. For nurses, the core competencies in disease and comorbidity management and function optimisation are related to “patient care” and those in teamwork are associated with “interpersonal and communication skills”.

The 2014 geriatric medicine milestones predominantly focused on internal medicine subspecialties and were not specifically tailored to geriatrics. However, the 2021 geriatric medicine Milestones 2.0 [8] were revised to reflect geriatrics-specific learning objectives, emphasizing geriatric knowledge and skills, interdisciplinary teamwork, and care within the context of patient values and family/caregiver needs. However, our study indicates that the majority of the identified geriatric core competencies still focus on subspecialty-specific milestones, particularly in patient care and medical knowledge, with less emphasis on interdisciplinary teamwork and patient-centered care. Considering the Milestone 2.0 framework, there appears to be a pressing need to redesign the geriatric curriculum to cover these critical core competencies more comprehensively, especially with regard to interdisciplinary team engagement. The current focus on sub-specialty milestones in geriatric education has created a gap between medical training and geriatric care. This approach prepares practitioners for subspecialty care, but fails to prepare them for the complexity of caring for older adults. Interdisciplinary teamwork is not just a complementary skill, but a foundational requirement in geriatric care. Collaborative efforts among different disciplines are essential in the context of fragmented systems.

Recently, Dimitriadou et al. [28] conducted a scoping review that specifically examined the competencies and skills required by primary care nurses to conduct comprehensive geriatric assessments (CGA). Their review synthesized 19 studies and identified six competency domains: clinical assessment, care planning and coordination, interpersonal skills, environmental and systemic competencies, technical and procedural competencies, quality improvement, and evidence-based practices. While their framework provides valuable insights into nursing-specific competencies for CGA in primary care settings, our review differs in both scope and analytic approaches. We included multi-professional groups (medical students, residents, practicing physicians, and nurses) and categorized 257 competencies across 18 domains using Bloom’s taxonomy and the ACGME competency framework. By applying a taxonomical lens, we not only mapped the frequency of competencies across studies but also highlighted their cognitive and knowledge-level distribution. This broader perspective complements the work of Dimitriadou et al. [28] by extending the discussion from nursing-specific CGA competencies to a more comprehensive, multi-professional perspective, underscoring the need for interdisciplinary curricula and competency development in geriatric education.

Additionally, Bloom’s taxonomy can serve as a useful framework for understanding and analysing the geriatric core competencies under review. In the original version of Bloom’s taxonomy (1956) [17], cognitive processes were classified into *Knowledge*, *Comprehension*, *Application*, *Analysis*, *Synthesis*, and *Evaluation*. In the revised version (2001) [18], they are classified as *Remember*, *Understand*, *Apply*, *Analyze*, *Evaluate*, and *Create*, with *Knowledge* forming the basis for these processes. *Knowledge* is further divided into factual, conceptual, procedural, and metacognitive categories.

Bloom’s taxonomy has been applied to the analysis of competency-based osteopathic medical education [29], but not to the evaluation of the growing need for geriatric core competencies. In the present study, 59.14% (152/257) of geriatric core competencies were classified as *Apply* in the cognitive process dimension. The prevalence of competencies in the *Apply* category suggests that learners are expected to move beyond information recall and comprehension by applying their knowledge to practical and problem-solving scenarios. Moreover, 64.2% (165/257) of the core competencies were classified as *Conceptual Knowledge* in the knowledge dimension. The substantial representation of *Conceptual Knowledge* in these competencies indicates that deep learning is essential for advanced learning and real-world applications.

Our findings have several important implications for future studies. The identified geriatric core competencies emphasized disease and comorbidity management, medication management, and cognitive problems; however, critical domains, such as interdisciplinary teamwork, caregiver engagement, and patient-centered care, were underrepresented. Addressing these gaps is essential to align educational curricula with the realities of geriatric care. Integrating these competencies into training pathways for medical students, residents, and nurses can support the development of more holistic and patient-centered approaches, while also strengthening collaboration across disciplines. In practice, this knowledge provides a foundation for curriculum redesign, faculty development, and competency-based assessments to better prepare healthcare professionals to meet the complex needs of older adults.

This review had several methodological limitations. Although a comprehensive search across five databases was conducted, only ten studies, mostly published before 2017, met the inclusion criteria. This relatively small number of included studies is partly attributable to our deliberate focus on research that employed structured consensus methods, such as Delphi, as these approaches provide clearer methodological rigor and enhance comparability across studies. Furthermore, we restricted the eligibility criteria to English-language publications, which may have excluded relevant studies published in other languages, contributing to the limited pool of evidence.

In addition, this review was conducted as a scoping review, which inherently emphasizes mapping key concepts and identifying trends in the existing literature rather than providing an exhaustive synthesis of all available evidence. Consequently, some recent developments and relevant grey literature may not have been captured. Finally, as most of the included studies originated in the United States and Europe, the generalizability of our findings to other cultural and educational contexts remains limited. Future research that employs a more comprehensive systematic review and meta-analysis is necessary to provide a more complete synthesis and quantitative assessment of the evidence.

## Conclusions

In conclusion, the most frequently mentioned geriatric core competencies were disease and comorbidity, pharmacologic/medication management, and cognitive/dementia problems, which belong to “patient care”, as proposed by ACGME. However, other crucial competencies, such as interdisciplinary teamwork, care within the context of patient values, and family/caregiver needs, have received less attention. This disparity underscores the need for a comprehensive revaluation of the geriatric curriculum. Integration of these less-discussed but vital competencies into the training of medical students, physicians, nurses, and other healthcare professionals is essential. This will ensure a more holistic approach to geriatric care, which is crucial for the field and beneficial to patient outcomes.

## Supplementary Information

Supplementary Material 1

Supplementary Material 2

## Data Availability

The datasets used during the current study are available from the corresponding author on reasonable request.

## Abbreviations

ACGME: Accreditation Council for Graduate Medical Education
AGS: American Geriatrics Society
CBME: Competency-based medical education
CFPC: College of Family Physicians of Canada
COE: Care of the elderly
ECOP: Educational Council of Osteopathic Principles
EM: Emergency medicine
Embase: Excerpta Medica database
FM: Family medicine
IM: Internal medicine
N/A: Not applicable
PGY: Post-graduate year
