# Supplementary material for: Geriatric core competencies for non-geriatricians and nurses: a scoping review
Source: BMC Geriatr. 2025 Nov 25;25:1054. doi: 10.1186/s12877-025-06806-8 (PMC12751425; doi:10.1186/s12877-025-06806-8)
Supplement: Supplementary file 2 — Supplementary Material 2 [file 12877_2025_6806_MOESM2_ESM.docx]

**Search Strategy**

| Database | # | Search syntax | Citations found |
| --- | --- | --- | --- |
| Embase | 1 | (nurse* OR (nursing NEAR/6 (staff* OR employee* OR officer* OR personnel* OR practitioner* OR profess* OR provider* OR specialist* OR worker*))):ti,kw | 185,926 |
|  | 2 | "nursing staff"/exp/mj OR "nurse"/exp/mj OR "nursing student"/exp/mj | 162,455 |
|  | 3 | Physician* OR Doctor* OR Surgeon* OR Residen* OR Internship* OR Internist* OR Intern OR Interns OR Clerk* OR (Medical NEAR/6 (student* OR undergraduate* OR graduate* OR postgraduate*)):ti,kw | 2,963,292 |
|  | 4 | Physicians/exp/mj OR "Students, Medical"/exp/mj OR "Internship and Residency"/exp/mj | 466,777 |
|  | 5 | (aged OR aging OR elder* OR geriatric* OR gerontolog* OR senior* OR senium* OR "old age*" OR (older Near/4 (adult* OR person* OR people OR patient* OR inpatient* OR outpatient* OR population* OR men OR women OR male OR female OR subject* OR citizen*))):ti,kw | 723,371 |
|  | 6 | "Geriatrics"/exp/mj OR "Gerontology"/exp/mj OR "aged"/exp/mj OR "geriatric disorder"/exp/mj OR "elderly care"/exp/mj OR "senior center"/exp/mj OR "aged hospital patient"/exp/mj | 211,259 |
|  | 7 | (competenc* OR abilit* OR aptitude* OR skill* OR knowledge* OR milestone* OR "mile stone*"):ti,kw | 338,397 |
|  | 8 | "competence"/exp/mj OR "aptitude"/exp/mj OR "skill"/exp/mj OR "knowledge"/exp/mj | 161,233 |
|  | 9 | ((#1 OR #2) OR (#3 OR #4) AND (#5 OR #6) AND (#7 OR #8)) AND [embase]/lim | 1,658 |
|  | 10 | #9 AND [2025-07-31]/sd | 11 |
| MEDLINE (Ovid) | 1 | (nurse* OR (nursing ADJ6 (staff* OR employee* OR officer* OR personnel* OR practitioner* OR profess* OR provider* OR specialist* OR worker*))).ti,kf | 174,845 |
|  | 2 | exp *"Nursing Staff"/ OR exp *"Nurses"/ OR exp *"Students, Nursing"/ | 158,177 |
|  | 3 | Physician* OR Doctor* OR Surgeon* OR Residen* OR Internship* OR Internist* OR Intern OR Interns OR Clerk* OR (Medical ADJ6 (student* OR undergraduate* OR graduate* OR postgraduate*)).ti,kf | 1,358,059 |
|  | 4 | exp *"Physicians"/ OR exp *"Students, Medical"/ OR exp *"Internship and Residency"/ | 204,288 |
|  | 5 | (aged OR aging OR elder* OR geriatric* OR gerontolog* OR senior* OR senium* OR "old age*" OR (older ADJ4 (adult* OR person* OR people OR patient* OR inpatient* OR outpatient* OR population* OR men OR women OR male OR female OR subject* OR citizen*))).ti,kf | 560,146 |
|  | 6 | exp *"Geriatrics"/ OR exp *"Aged"/ OR exp *"Senior Centers"/ | 56,108 |
|  | 7 | (competenc* OR abilit* OR aptitude* OR skill* OR knowledge* OR milestone* OR "mile stone*").ti,kf | 274,775 |
|  | 8 | exp *"Professional Competence"/ OR exp *"Aptitude"/ OR exp *"Knowledge"/ | 75,274 |
|  | 9 | (1 OR 2 OR 3 OR 4) AND (5 OR 6) AND (7 OR 8) | 2,103 |
|  | 10 | 9 and (20231124:20250731).ed. | 0 |
| 3)Cochrane CENTRAL | 1 | (nurse* OR (nursing NEAR/5 (staff* OR employee* OR officer* OR personnel* OR practitioner* OR profess* OR provider* OR specialist* OR worker*))):ti,kw | 16,808 |
|  | 2 | [mh "Nursing Staff"[mj]] OR [mh "Nurses"[mj]] OR [mh "Students, Nursing"[mj]] | 1,643 |
|  | 3 | Physician* OR Doctor* OR Surgeon* OR Residen* OR Internship* OR Internist* OR Intern OR Interns OR Clerk* OR (Medical NEAR/5 (student* OR undergraduate* OR graduate* OR postgraduate*)):ti,kw | 142,130 |
|  | 4 | [mh "Physicians"[mj]] OR [mh "Students, Medical"[mj]] OR [mh "Internship and Residency"[mj]] | 2,877 |
|  | 5 | (aged OR aging OR elder* OR geriatric* OR gerontolog* OR senior* OR senium* OR (old NEXT age*) OR (older NEAR/3 (adult* OR person* OR people OR patient* OR inpatient* OR outpatient* OR population* OR men OR women OR male OR female OR subject* OR citizen*))):ti,kw | 588,086 |
|  | 6 | [mh "Geriatrics"[mj]] OR [mh "Aged"[mj]] OR [mh "Senior Centers"[mj]] | 430 |
|  | 7 | (competenc* OR abilit* OR aptitude* OR skill* OR knowledge* OR milestone* OR "mile stone*"):ti,kw | 44,071 |
|  | 8 | [mh "Professional Competence"[mj]] OR [mh "Aptitude"[mj]] OR [mh "Knowledge"[mj]] | 1,250 |
|  | 9 | (#1 OR #2 OR #3 OR #4) AND (#5 OR #6) AND (#7 OR #8)  Limits in: 20231124-20250731 | 95 |
| Ageline  (EBSCOhost) | 1 | TI ( (nurse* OR (nursing N4 (staff* OR employee* OR officer* OR personnel* OR practitioner* OR profess* OR provider* OR specialist* OR worker*)) | 8,778 |
|  | 2 | (DE "Nurses" OR DE "Visiting Nurses") OR (DE "Nurse Practitioners") OR (DE "Nurses Aides") | 2,553 |
|  | 3 | TI ( Physician* OR Doctor* OR Surgeon* OR Residen* OR Internship* OR Internist* OR Intern OR Interns OR Clerk* OR (Medical N4 (student* OR undergraduate* OR graduate* OR postgraduate*) ) | 35,699 |
|  | 4 | DE (physicians) | 1,920 |
|  | 5 | TI (aged OR aging OR elder* OR geriatric* OR gerontolog* OR senior* OR senium* OR "old age*" OR (older N2 (adult* OR person* OR people OR patient* OR inpatient* OR outpatient* OR population* OR men OR women OR male OR female OR subject* OR citizen*))) | 87,840 |
|  | 6 | (DE "Geriatrics" OR DE "Gerontological Nursing") OR (DE "Gerontology") OR (DE "Older Adults") | 12,541 |
|  | 7 | TI (competenc* OR abilit* OR aptitude* OR skill* OR knowledge* OR milestone* OR "mile stone*") | 4,707 |
|  | 8 | (DE "Legal Competency") OR (DE "Knowledge Level") | 256 |
|  | 9 | (S1 OR S2 OR S3 OR S4) AND (S5 OR S6) AND (S7 OR S8) | 345 |
|  | 10 | S9 AND DT 20231124-20250731 | 18 |
| 1. ERIC   (EBSCOhost) | 1 | TI ( (nurse* OR (nursing N4 (staff* OR employee* OR officer* OR personnel* OR practitioner* OR profess* OR provider* OR specialist* OR worker*)) | 4,985 |
|  | 2 | (DE "Nurses" OR DE "Visiting Nurses") OR (DE "Nurse Practitioners") OR (DE "Nurses Aides") | 3,711 |
|  | 3 | TI ( Physician* OR Doctor* OR Surgeon* OR Residen* OR Internship* OR Internist* OR Intern OR Interns OR Clerk* OR (Medical N4 (student* OR undergraduate* OR graduate* OR postgraduate*) ) | 74,820 |
|  | 4 | DE (physicians) | 4,437 |
|  | 5 | TI (aged OR aging OR elder* OR geriatric* OR gerontolog* OR senior* OR senium* OR "old age*" OR (older N2 (adult* OR person* OR people OR patient* OR inpatient* OR outpatient* OR population* OR men OR women OR male OR female OR subject* OR citizen*))) | 14,988 |
|  | 6 | ((DE "Geriatrics" OR DE "Gerontological Nursing") OR (DE "Gerontology")) OR (DE "Older Adults") | 16,311 |
|  | 7 | TI (competenc* OR abilit* OR aptitude* OR skill* OR knowledge* OR milestone* OR "mile stone*") | 89,799 |
|  | 8 | (DE "Legal Competency") OR (DE "Knowledge Level") | 23,913 |
|  | 9 | (S1 OR S2 OR S3 OR S4) AND (S5 OR S6) AND (S7 OR S8) | 167 |
|  | 10 | S9 AND DT 20231124-20250731 | 6 |

Notes:

The initial search was conducted in June 2022. The first update was performed in November 2023, and the most recent update was completed in July 2025. The strategies reported above reflect the final search update.
